# Inner and outer retinal layer thickness alterations in pediatric and juvenile craniopharyngioma

**DOI:** 10.1038/s41598-021-82107-5

**Published:** 2021-02-02

**Authors:** Ga-In Lee, Kyung-Ah Park, Sei Yeul Oh, Doo-Sik Kong, Sang Duk Hong

**Affiliations:** 1 Department of Ophthalmology, Samsung Medical Center, Sungkyunkwan University School of Medicine, 81 Irwon-ro, Gangnam-gu, Seoul, 06351 South Korea; 2 Department of Neurosurgery, Endoscopic Skull Base Surgery Clinic, Brain Tumor Center, Samsung Medical Center, Sungkyunkwan University School of Medicine, Seoul, South Korea; 3 Department of Otorhinolaryngology-Head and Neck Surgery, Samsung Medical Center, Sungkyunkwan University School of Medicine, Seoul, South Korea

**Keywords:** Biomarkers, Neurology

## Abstract

We evaluated postoperative retinal thickness in pediatric and juvenile craniopharyngioma (CP) patients with chiasmal compression using optical coherence tomography (OCT) auto-segmentation. We included 18 eyes of 18 pediatric or juvenile patients with CP and 20 healthy controls. Each thickness of the macular retinal nerve fiber layer (RNFL), ganglion cell layer (GCL), inner plexiform layer (IPL), inner nuclear layer, outer plexiform layer, outer nuclear layer, and photoreceptor layer was compared between the CP patients and healthy controls. There was significant thinning in the macular RNFL (estimates [μm], superior, − 10.68; inferior, − 7.24; nasal, − 14.22), all quadrants of GCL (superior, − 16.53; inferior, − 14.37; nasal, − 24.34; temporal, − 9.91) and IPL (superior, − 11.45; inferior, − 9.76; nasal, − 15.25; temporal, − 4.97) in pediatric and juvenile CP patients postoperatively compared to healthy control eyes after adjusting for age and refractive errors. Thickness reduction in the average and nasal quadrant of RNFL, GCL, and IPL was associated with peripapillary RNFL thickness, and reduced nasal quadrant GCL and IPL thicknesses were associated with postoperative visual field defects. In pediatric and juvenile patients with CP, decreased inner retinal layer thickness following chiasmal compression was observed. The changes in retinal structures were closely related to peripapillary RNFL thinning and functional outcomes.

## Introduction

Craniopharyngioma (CP) is a rare benign tumor originating from the remnants of Rathke’s pouch, with a bimodal age distribution of 5–14 years and 50–75 years^1–3^. It is the most common suprasellar tumor in childhood and the second most common tumor following pituitary adenoma in adults^4^. Although it is classified as a benign tumor, CP often exhibits aggressive behavior^5^. Because of the anatomical closeness of CP to critical structures, including the pituitary, hypothalamus, and visual apparatus, pediatric CP is one of the most challenging childhood brain tumors^6,7^. Consequently, long-term survivors live independently but have a lower overall quality of life as a consequence of visual and hypothalamic dysfunction^8^.

Because of the proximity to visual structures, CP may cause severe damage to the optic nerve, with corresponding visual field (VF) defects or loss of visual acuity. As a consequence, patients with pediatric CP usually present with visual complaints. However, young children are frequently unable to complete visual function testing due to a lack of cooperation, and it is difficult to evaluate visual dysfunction related to CP^9^. Optical coherence tomography (OCT) measurements have been suggested as a parameter that indirectly reflects visual function in children with tumors involving the optic pathway^9,10^. OCT enables structural imaging of the retina with a resolution higher than any other non-invasive imaging modality^11^. It is sensitive to spatial variations in the optical reflectivity or scattering of tissue associated with local changes in the refractive index or structure^11^. OCT is reported to be useful in diagnosing and predicting the prognosis of adult patients with brain tumors affecting the visual pathway^12^. Tieger et al. reported that nasal ganglion cell layer complex (GCC) loss seen on OCT might be a useful marker for detecting chiasmal compression due to brain tumors^12^.

There are several normative OCT databases for the pediatric population^13–15^. Studies on OCT in children with visual loss have also proved the reproducibility of OCT measurements^16,17^. The usefulness of OCT in children with tumors compressing the optic nerve or optic chiasms, such as optic pathway glioma^18,19^ and CP^10,20^, was reported previously. Avery et al. investigated the relationship between visual acuity and RNFL thickness in patients with optic pathway glioma and suggested that RNFL thickness might be useful as an indicator of visual function in patients who are unable to cooperate with visual acuity testing^18^. Gu et al. reported that GCC thickness measured by OCT could distinguish between patients with and without a loss of visual function from optic pathway glioma^19^. Yang et al. studied OCT results in eyes with CP and reported that the GCC thickness was sensitive in detecting optic nerve injury especially in pediatric or juvenile patients^20^. Bialer et al. also reported that a thinner RNFL on OCT was well-correlated with poorer visual acuity and VF defects in pediatric CP^10^. However, there have been no studies on changes in the whole retinal layer using retinal segmentation analysis in pediatric and juvenile patients with CP. In this study, we evaluated changes in both the inner and outer retinal layer thicknesses in pediatric and juvenile patients with CP compared to healthy controls using OCT auto-segmentation software and analyzed the association between retinal layer thickness and other ocular structural and functional parameters.

## Results

This study included a total of 18 eyes of 18 patients with pediatric and juvenile CP and 20 eyes of 20 healthy controls. Except for only one patient with papillary type CP, all patients were diagnosed with adamantinomatous CPs histologically. The mean age was 11 ± 4 years in patients with CP, and 10 ± 4 years in the healthy controls (*p* = 0.3310). There were significant differences in visual acuity between the two groups (*p* = 0.0360). Also, there was a significant thinning of the peripapillary RNFL in the patient group compared to the healthy controls (*p* < 0.0001). In the patient group, 12 patients had permanent hemianopsia with visual acuity greater than 20/40, and two patients had permanent loss of both the VF and visual acuity after tumor resection. VF testing was not performed in four patients due to poor cooperation (Table 1).

**Table 1:** Demographics of postoperative pediatric and juvenile craniopharyngioma patients and healthy controls.

	Patients (N = 18)	Controls (N = 20)	*p*-value
Age (years)	11 ± 4	10 ± 4	0.3310*
Sex (M/F)	9/9	14/6	0.3200^†^
Spherical equivalent	− 1.60 ± 2.11	− 0.77 ± 2.13	0.1410*
VF (MD, dB)	− 14.15 ± 7.06 [− 30.58, − 2.29]	NA	
logMAR BCVA	0.13 ± 0.20	0.02 ± 0.07	0.0360*
pRNFL thickness (μm)	56.81 ± 13.60	84.05 ± 9.68	< 0.0001*

Significant values are indicated in bold.

*N* numbers, *dB* decibel, *VF* visual field, *MD* mean deviation, *BCVA* best corrected visual acuity, *pRNFL* peripapillary retinal nerve fiber layer, *NA* not applicable.

*Mann–Whitney U test.

^†^Fisher’s exact test.

Table 2 presents the results of each retinal layer thickness in four quadrants of the two groups. A significant difference was observed in almost all quadrants of the macular RNFL (estimates (standard error), superior, − 10.68 (1.46), *p* < 0.0001; inferior, − 7.24 (1.20), *p* < 0.0001; nasal, − 14.22 (1.20), *p* < 0.0001), ganglion cell layer (GCL) (superior, − 16.53 (1.50), *p* < 0.0001; inferior, − 14.37 (1.52), *p* < 0.0001; nasal, − 24.34 (1.40), *p* < 0.0001; temporal, − 9.91 (2.24), *p* = 0.0026), and inner plexiform layer (IPL) (superior, − 11.45 (0.94), *p* < 0.0001; inferior, − 9.76 (0.91), *p* < 0.0001; nasal, − 15.25 (0.82), *p* < 0.0001; temporal, − 4.97 (1.12), *p* = 0.0024) between the patients with CP and the healthy controls after adjusting for age and spherical equivalent (SE). No significant difference was detected in the inner nuclear layer (INL), outer plexiform layer (OPL), outer nuclear layer (ONL), or photoreceptor layer (PRL) thickness between the two groups.

**Table 2:** Comparison of intraretinal layer thickness between pediatric and juvenile craniopharyngioma patients and healthy controls.

	Patients (N = 18)	Controls (N = 20)	Estimates	Standard error	p-value*
**Superior quadrant (μm)**					
RNFL	21.61 ± 5.55	32.03 ± 2.75	− **10.677**	**1.456**	** < 0.0001**
GCL	29.61 ± 6.08	46.60 ± 2.51	− **16.533**	**1.504**	** < 0.0001**
IPL	25.69 ± 3.16	37.38 ± 2.42	− **11.446**	**0.935**	** < 0.0001**
INL	41.89 ± 4.33	39.05 ± 3.32	3.364	1.202	0.2348
OPL	35.25 ± 9.57	34.70 ± 8.17	1.907	2.812	1.0000
ONL	61.36 ± 12.61	60.43 ± 10.87	0.966	3.975	1.0000
PRL	67.44 ± 2.00	67.03 ± 2.45	0.561	0.741	1.0000
**Inferior quadrant (μm)**					
RNFL	23.72 ± 4.75	30.73 ± 1.95	− **7.238**	**1.198**	** < 0.0001**
GCL	31.94 ± 5.99	46.58 ± 2.49	− **14.373**	**1.523**	** < 0.0001**
IPL	26.75 ± 3.30	36.88 ± 2.25	− **9.761**	**0.908**	** < 0.0001**
INL	39.86 ± 4.71	38.88 ± 3.28	1.567	1.239	1.0000
OPL	30.42 ± 4.33	30.78 ± 5.54	− 0.220	1.690	1.0000
ONL	60.25 ± 10.32	60.3 ± 8.40	0.852	3.094	1.0000
PRL	66.11 ± 2.83	66.3 ± 2.86	0.020	0.957	1.0000
**Nasal quadrant (μm)**					
RNFL	19.89 ± 3.98	34.05 ± 3.06	− **14.223**	**1.202**	** < 0.0001**
GCL	22.92 ± 5.48	47.33 ± 2.32	− **24.338**	**1.404**	** < 0.0001**
IPL	22.14 ± 3.15	37.50 ± 1.44	− **15.250**	**0.816**	** < 0.0001**
INL	40.86 ± 4.33	38.03 ± 2.84	3.332	1.155	0.1894
OPL	30.53 ± 3.37	29.53 ± 3.44	1.191	1.140	1.0000
ONL	67.53 ± 9.94	66.48 ± 6.27	1.786	2.686	1.0000
PRL	68.31 ± 2.12	67.88 ± 2.28	0.572	0.727	1.0000
**Temporal quadrant (μm)**					
RNFL	17.22 ± 1.45	17.35 ± 1.03	− 0.135	0.420	1.0000
GCL	34.31 ± 9.16	44.45 ± 2.66	− **9.914**	**2.241**	**0.0026**
IPL	32.33 ± 4.44	37.55 ± 1.90	− **4.975**	**1.116**	**0.0024**
INL	36.61 ± 2.39	37.93 ± 2.48	− 0.816	0.669	1.0000
OPL	30.11 ± 4.59	30.53 ± 3.30	0.256	1.192	1.0000
ONL	61.78 ± 10.94	62.98 ± 6.54	− 0.884	3.026	1.0000
PRL	67.00 ± 2.57	67.65 ± 2.45	− 0.409	0.829	1.0000

Significant values are indicated in bold.

*N* numbers, *RNFL* retinal nerve fiber layer, *GCL* ganglion cell layer, *IPL* inner plexiform layer, *INL* inner nuclear layer, *OPL* outer plexiform layer, *ONL* outer nuclear layer, *PRL* photoreceptor layer.

*P-values obtained by linear regression were adjusted for age and SE and Bonferroni’s correction multiplied by 28.

To compare the retinal layer thickness between the eye with better visual function and the eye with poor visual function in the CP patients, we performed a subgroup analysis. Table 3 presents the measurements of each retinal layer thickness in four quadrants of the worse eye (worse VF) and better eye (better VF) in the CP patients compared to the controls. The mean thickness of the RNFL, GCL, and IPL was less in the worse eye group than in the better eye group. However, the differences did not reach statistical significance. Both the better eye and worse eye groups showed significant differences in RNFL, GCL, and IPL thickness compared to the control group in most sectors (all *p* < 0.05).

**Table 3:** Comparison of intraretinal layer thickness between worse and better eye in pediatric and juvenile craniopharyngioma patients and healthy controls.

	Worse eye (N = 16)	Better eye (N = 16)	Controls (N = 20)	*p*-value (worse vs better)	*p*-value (worse vs control)	*p*-value (better vs control)
**Superior quadrant (μm)**						
RNFL	19.03 ± 5.58	23.59 ± 5.03	32.03 ± 2.75	0.2598*	< 0.0001*	** < 0.0001**^**‡**^
GCL	27.72 ± 6.82	33.13 ± 7.54	46.60 ± 2.51	1.0000^†^	** < 0.0001**^**‡**^	** < 0.0001**^**‡**^
IPL	25.00 ± 3.85	27.94 ± 4.15	37.38 ± 2.42	1.0000^†^	** < 0.0001**^**‡**^	** < 0.0001**^**‡**^
INL	41.44 ± 4.42	40.88 ± 4.41	39.05 ± 3.32	1.0000^†^	1.0000^‡^	1.0000^‡^
OPL	34.78 ± 6.97	33.47 ± 8.03	34.70 ± 8.17	1.0000^†^	1.0000*	1.0000*
ONL	60.50 ± 12.44	60.22 ± 11.90	60.43 ± 10.87	1.0000^†^	1.0000^‡^	1.0000^‡^
PRL	67.28 ± 2.86	67.03 ± 2.69	67.03 ± 2.45	1.0000*	1.0000^‡^	1.0000^‡^
**Inferior quadrant (μm)**						
RNFL	21.19 ± 6.05	25.50 ± 4.51	30.73 ± 1.95	0.2582^†^	** < 0.0001**^**‡**^	**0.0015**^**‡**^
GCL	28.53 ± 6.44	34.84 ± 6.39	46.58 ± 2.49	0.1702^†^	< 0.0001*	< 0.0001*
IPL	25.00 ± 3.85	27.94 ± 4.15	36.88 ± 2.25	0.1272^†^	** < 0.0001**^**‡**^	** < 0.0001**^**‡**^
INL	40.91 ± 5.20	38.28 ± 3.11	38.88 ± 3.28	0.5905^†^	1.0000*	1.0000*
OPL	30.28 ± 6.47	30.31 ± 4.77	30.78 ± 5.54	1.0000*	1.0000*	1.0000*
ONL	58.03 ± 8.12	58.69 ± 9.29	60.3 ± 8.40	1.0000^†^	1.0000^‡^	1.0000^‡^
PRL	66.47 ± 3.27	66.28 ± 2.73	66.3 ± 2.86	1.0000*	1.0000*	1.0000*
**Nasal quadrant (μm)**						
RNFL	18.72 ± 4.22	22.06 ± 5.36	34.05 ± 3.06	0.8604^†^	** < 0.0001**^**‡**^	** < 0.0001**^**‡**^
GCL	23.59 ± 6.91	26.03 ± 8.69	47.33 ± 2.32	1.0000*	< 0.0001*	< 0.0001*
IPL	22.16 ± 4.40	23.59 ± 5.75	37.50 ± 1.44	1.0000*	< 0.0001*	< 0.0001*
INL	41.06 ± 3.30	39.56 ± 4.08	38.03 ± 2.84	1.0000^†^	0.1521^‡^	1.0000^‡^
OPL	30.81 ± 3.82	29.66 ± 2.95	29.53 ± 3.44	1.0000^†^	1.0000^‡^	1.0000^‡^
ONL	64.94 ± 8.33	65.53 ± 8.29	66.48 ± 6.27	1.0000^†^	1.0000^‡^	1.0000^‡^
PRL	68.09 ± 2.73	67.94 ± 2.63	67.88 ± 2.28	1.0000^†^	1.0000^‡^	1.0000^‡^
**Temporal quadrant (μm)**						
RNFL	16.66 ± 1.86	17.16 ± 1.31	17.35 ± 1.03	1.0000*	1.0000*	1.0000^‡^
GCL	28.59 ± 9.93	37.59 ± 8.06	44.45 ± 2.66	0.3649*	** < 0.0001**^**‡**^	0.0094*
IPL	28.44 ± 5.84	33.28 ± 4.29	37.55 ± 1.90	0.2092^†^	** < 0.0001**^**‡**^	0.0082*
INL	36.84 ± 2.91	36.06 ± 2.11	37.93 ± 2.48	1.0000^†^	1.0000^‡^	1.0000^‡^
OPL	28.81 ± 2.91	30.22 ± 4.54	30.53 ± 3.30	1.0000*	1.0000*	1.0000*
ONL	63.22 ± 7.88	58.94 ± 9.33	62.98 ± 6.54	0.2300^†^	1.0000^‡^	1.0000^‡^
PRL	67.53 ± 3.09	66.84 ± 2.73	67.65 ± 2.45	1.0000*	1.0000^‡^	1.0000^‡^

Significant values are indicated in bold.

*N* numbers, *RNFL* retinal nerve fiber layer, *GCL* ganglion cell layer ganglion cell layer, *IPL* inner plexiform layer, *INL* inner nuclear layer, *OPL* outer plexiform layer, *ONL* outer nuclear layer, *PRL* photoreceptor layer.

*P-values obtained by the Wilcoxon signed rank test and Bonferroni’s correction multiplied by 28.

^†^*P*-values obtained by the paired t-test and Bonferroni’s correction multiplied by 28.

^‡^*P*-values obtained by T-test and Bonferroni’s correction multiplied by 28.

**Figure 1:**
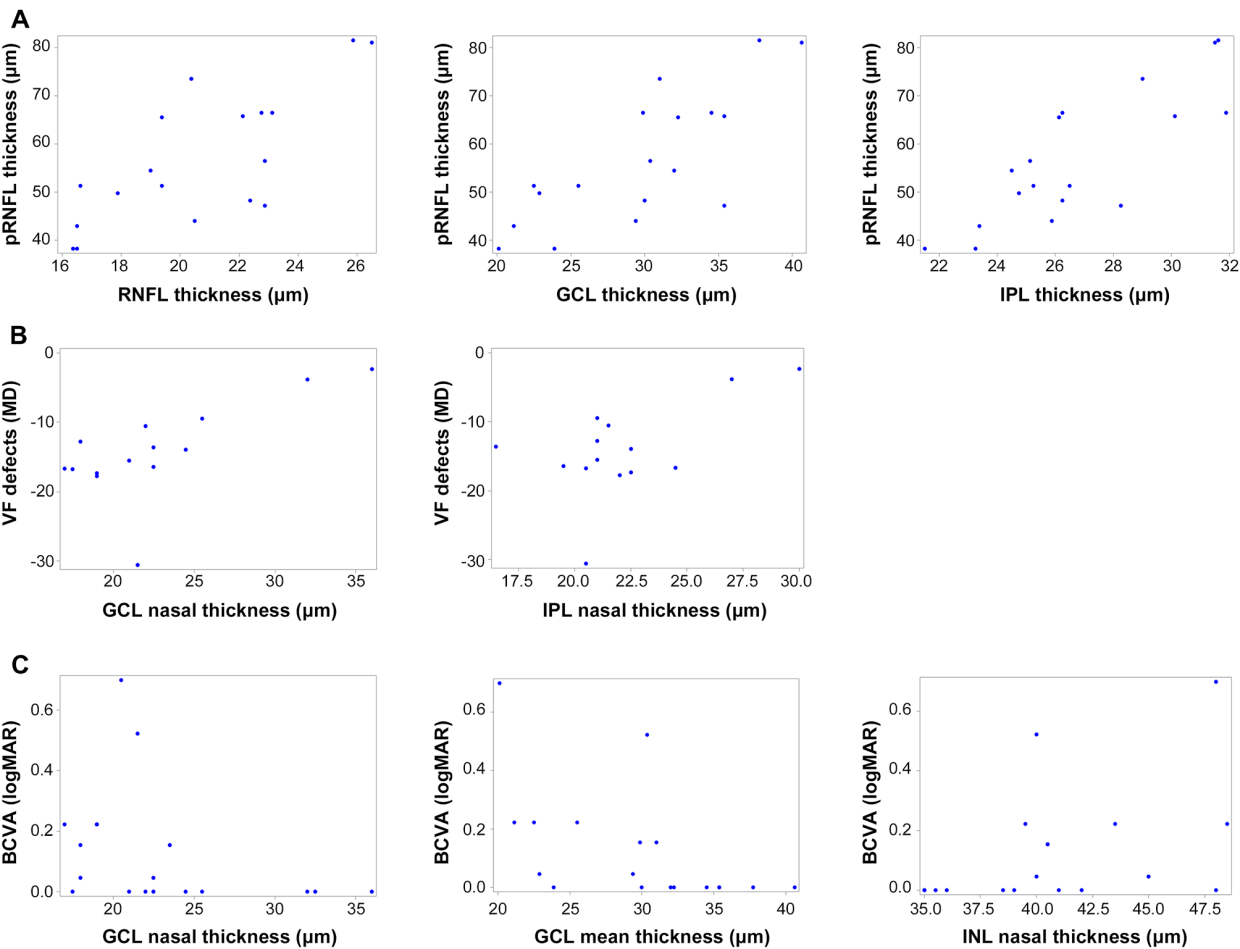
Scatter plots show the association between postoperative intra-retinal layer thickness and other ocular parameters including peripapillary retinal nerve fiber layer thickness (pRNFL), visual field (VF) defects, and best-corrected visual acuity (BCVA). The associations between (**A**) postoperative pRNFL thickness and the macular RNFL (*r* = 0.7450, p = 0.0004), GCL (*r* = 0.7739, *p* = 0.0002), and IPL (*r* = 0.8257, *p* < 0.0001) are presented. (**B**) Postoperative VF defects were associated with nasal quadrant GCL (*r* = 0.6806, *p* = 0.0074) and IPL (*r* = 0.5662, *p* = 0.0348) thickness, and (**C**) the postoperative BCVA was associated with the average value of GCL and nasal quadrant GCL (*r* = − 0.7162, *p* = 0.0008; *r* = − 0.5413, *p* = 0.0203) and INL (*r* = 0.4962, *p* = 0.0362) thickness.

Table 4 and Fig. 1 present the associations between postoperative intraretinal layer thickness and other ocular parameters. Peripapillary RNFL thickness was associated with macular RNFL (*r* = 0.7450, *p* = 0.0004), GCL (*r* = 0.7739, *p* = 0.0002), and IPL (*r* = 0.8257, *p* < 0.0001) thickness. Regarding the functional parameters, postoperative VF defects were associated with the thickness of the nasal quadrant GCL (*r* = 0.6806, *p* = 0.0074) and IPL (*r* = 0.5662, *p* = 0.0348). The postoperative best-corrected visual acuity (BCVA) was also associated with the average value of GCL, nasal quadrant GCL (*r* = − 0.7162, *p* = 0.0008; *r* = − 0.5413, *p* = 0.0203) and nasal quadrant INL (r = 0.4962, *p* = 0.0362) thickness.

**Table 4:** Association analysis of postoperative intra-retinal layer thickness versus visual field defects, visual acuity, and peripapillary retinal nerve fiber layer thickness.

Intra-retinal layers	pRNFL thickness		VF defects		BCVA	
	*r*	*p*-value	*r*	*p*-value	*r*	*p-*value
RNFL mean	**0.7450**	0.0004*	0.1607	0.5861*	− 0.3823	0.1174^†^
RNFL nasal	**0.6977**	0.0013*	0.2400	0.4086*	− 0.2306	0.3572^†^
GCL mean	**0.7739**	0.0002*	0.3487	0.2218*	− **0.7162**	**0.0008**^†^
GCL nasal	**0.5549**	**0.0168**^†^	**0.6806**	**0.0074**^†^	− **0.5413**	**0.0203**^†^
IPL mean	**0.8257**	< 0.0001*	0.4972	0.0705*	− 0.4345	0.0716^†^
IPL nasal	**0.5557**	0.0166*	**0.5662**	0.0348*	− 0.2035	0.4179^†^
INL mean	− 0.0515	0.8392*	− 0.2060	0.4799*	0.4331	0.0726^†^
INL nasal	− 0.1898	0.4507*	− 0.2868	0.3201*	**0.4962**	**0.0362**^†^
OPL mean	− 0.0311	0.9024*	− 0.1644	0.5743*	0.3869	0.1127^†^
OPL nasal	− 0.3623	0.1396*	− 0.2680	0.3542*	0.3940	0.1057^†^
ONL mean	0.1132	0.6548*	− 0.1949	0.5044*	0.2810	0.2586^†^
ONL nasal	0.2577	0.3019*	− 0.1892	0.5171*	0.1976	0.4319^†^
PRL mean	0.1692	0.5022^†^	0.1364	0.6419^†^	− 0.3013	0.2244^†^
PRL nasal	0.0417	0.8696*	0.1648	0.5734*	− 0.3783	0.1216^†^

Significant values are indicated in bold.

*RNFL* retinal nerve fiber layer, *pRNFL* peripapillary retinal nerve fiber layer, *VF* visual field, *MD* mean deviation, *BCVA* best-corrected visual acuity, *GCL* ganglion cell layer, *IPL* inner plexiform layer, *INL* inner nuclear layer, *OPL* outer plexiform layer, *ONL* outer nuclear layer, *PRL* photoreceptor layer.

*Pearson’s correlation analysis.

^†^Spearman’s correlation analysis.

## Discussion

Compared to pituitary adenoma, which is the most common type of sellar tumor in adults, CP is known to be deep-seated, with a midline location and intimate relationship with critical neurovascular structures, leading to increased struggles in surgical removal^21^. Mediero et al. reported that 60% of the eyes with CP had a BCVA below 20/40, and VF defects were more frequently observed than the loss of visual acuity after surgery^22^. Also, Wan et al. reported that 58% of the CP patients had visual impairment in at least one eye at the final follow-up^23^. In contrast, in pediatric patients with pituitary adenoma, only 6% of the pediatric patients presented with permanent visual dysfunction after surgery^24^. Also, the visual outcomes of patients with pediatric CP are reported to be poorer than those of adult CP. Chen et al. demonstrated that temporal hemianopsia was particularly predominant in pediatric patients who tended to present late because of the late detection of visual dysfunction^25^. They reported that adult CP patients tended to have more subtle VF changes, such as generalized field constriction and central scotoma^25^.

Retinal layer thinning in chiasmal compression or other optic neuropathies was reported to be caused by retrograde degeneration^26,27^. In animal studies, a complete loss of ganglion cells in the medial retina was found after axonal injury due to chiasmal compression^28^. This was also confirmed by OCT studies in adult patients with chiasmal compression^29,30^. Previous studies using OCT reported inner-retinal layer thinning following chiasmal compression^29,30^. Akashi et al. reported that macular inner retinal layer thinning in the nasal hemiretina had diagnostic value for band atrophy due to chiasmal compression^29^. Monteiro et al. also evaluated macular inner retinal layer thickness using OCT in eyes with band atrophy due to chiasmal compression^30^ and reported that band atrophy led to macular RNFL, GCL, and IPL thinning and INL thickening. And, segmented retinal layer analysis was suggested to be a useful tool for the assessment of structure–function relationships in patients with temporal VF defects^30^. Reports on the OCT results in pediatric tumors involving the visual pathway are relatively scarce compared to studies in the adult population, and there have been a few reported studies using OCT in pediatric patients with optic pathway glioma^9,18,19^ and CP^10,22^. Avery et al. reported that in optic pathway glioma, decreased RNFL thickness was correlated with low visual acuity or VF deficits^18^. Gu et al. also reported that GCC thickness had discriminatory ability between normal and abnormal vision in patients with optic pathway glioma^19^. Since the use of other visual functional testing is often limited in pediatric patients due to the lack of cooperation, Banc et al. suggested that OCT may serve as a potential tool for screening or follow-up examinations in children with optic pathway glioma^9^. In 20 pediatric patients with CP, Bialer et al. demonstrated peripapillary RNFL thinning and a significant correlation between peripapillary RNFL thickness and visual acuity and the VF^10^. They also demonstrated that OCT measurements might be successfully performed in children who are neurologically disabled^10^. Mediero et al. also reported that the GCC and RNFL thickness was significantly associated with VF defects in 10 pediatric patients with CP^22^. In addition, Yang et al. reported a comparison of GCC and peripapillary RNFL thickness between children and adults with CP^20^. The GCC thickness in the pediatric and juvenile CP patients was significantly thinner than that in the adult CP patients and reflected more severe retinal structural degeneration due to retrograde degeneration^28^ following axonal damage in pediatric and juvenile CP patients compared to adult CP patients^20^.

In this study, we first performed whole retinal layer segmentation analyses in pediatric and juvenile patients with CP. Previous studies in adult patients with chiasmal compression revealed pathologic changes in various retinal layers such as the INL^30^, IPL^31,32^, and PRL^31^ following chiasmal compression and significant association between the thinning of retinal layers and visual prognosis^29,32^. We also found a significant thinning of the RNFL, GCL, and IPL in pediatric and juvenile CP patients compared to healthy controls. Otherwise, no significant alterations were observed in the INL, OPL, ONL, and PRL in patients with CP. We also found that the nasal GCL and IPL thickness was associated with the degree of postoperative VF defects, which suggests that retinal layer thickness changes represent postoperative visual function in these subjects. Retinal layer segmentation analysis, especially including inner retinal layers such as the GCL and IPL, might be useful for the postoperative follow-up of pediatric and juvenile CP patients.

A number of important limitations should be considered when interpreting the data from our study. First, we used a retrospective cross-sectional design, thereby limiting our ability to reveal the temporal aspects of changes in the OCT parameters. Second, our dataset was relatively small and the patients were inconsistently followed after decompression surgery. This was uncontrollable with a retrospective design. Ultimately, a larger longitudinal cohort study is needed to observe the serial changes in intraretinal layer thicknesses. Third, since this was an exploratory study, and a primary analysis was not planned before the study, all results should be read as descriptive and interpreted with caution^33^.

In conclusion, this study revealed inner retinal layer degeneration in pediatric and juvenile CP patients with chiasmal compression. The nasal GCL and IPL thicknesses were associated with the degree of postoperative VF defects. These results suggest that retinal layer segmentation analysis using OCT may be a useful tool in estimating the degree of functional damage in pediatric CP patients.

## Methods

### Study participants

This retrospective case–control study included 18 patients with pediatric and juvenile CP and 20 healthy controls at the Neuro-ophthalmology Department of Samsung Medical Center between March 2012 and April 2020. This study was conducted according to the tenets of the Declaration of Helsinki and was approved by the Institutional Review Board of the Samsung Medical Center (Seoul, Republic of Korea, IRB No. 2018-06-085-003). Informed consent was waived for the patients with chiasmal compression by the Institutional Review Board of the Samsung Medical Center (Seoul, Republic of Korea). For all included patients, the clinical diagnosis of chiasmal compression with CP was based on magnetic resonance imaging (MRI) evidence of tumor compression of the optic chiasm and pathologically confirmed as CP postoperatively. All patients underwent a trans-sphenoidal approach or open craniotomy with tumor resection. Seven patients also received adjuvant radiotherapy. All patients were followed up for at least 6 months after surgery. The control group consisted of 4 to 17-year-old healthy volunteers who underwent routine eye examinations. Written informed consent was obtained from their legal guardians and, when appropriate, the healthy children, before enrollment. None of the controls had a history of ocular or neurologic disease. The healthy controls were required to have normal visual acuity, a normal intraocular pressure of ≤ 21 mmHg, and normal optic discs. The following categories of patients or healthy controls were excluded: those diagnosed with other ophthalmic diseases (glaucoma, a refractive error greater than + 6.0 diopters of SE or less than − 6.0 diopters of spherical equivalent, astigmatism of 3.0 diopters or more, amblyopia, epiretinal membrane, age-related macular degeneration, diabetic retinopathy, retinal artery/vein occlusion, optic neuritis, or other ischemic optic neuropathy) and previous retinal surgery that affected the thickness of the intra-retinal layer, and those diagnosed with known systematic/inflammatory diseases such as cancer and multiple sclerosis.

### Ophthalmic examinations

All subjects were scanned using fundus color photography and spectral-domain OCT (SD-OCT, Spectralis, Heidelberg Engineering, Heidelberg, Germany). The VF perimetry of the patients was measured with a Humphrey Field Analyzer using the 30-2 SITA-standard protocol (Humphrey 740 Visual Field Analyzer, Carl Zeiss Meditec Inc. Dublin, CA). Only reliable VFs (≤ 33% false positives and false negatives; fixation losses of < 20%) were used in the study. The mean deviation (MD) was used for analysis.

### OCT analysis and segmentation

In patients with chiasmal compression, all OCT imaging was performed postoperatively. In each patient, only a single eye showing the worse visual acuity or VF defects was selected for the main analysis in order to avoid biases resulting from inter-eye correlation. However, if visual acuity was less than 20/200 in the eye with worse VF defects, the other eye with visual acuity greater than 20/200 was selected for more accurate OCT examination with better cooperation. In healthy controls, only the right eye data were analyzed, except when the data quality of the right eye was inappropriate and the data quality of the left eye was appropriate for the analysis of retinal layer thickness. If the pupil diameter was not sufficient to obtain OCT images successfully, mydriatic eye drops (0.5% tropicamide and 0.5% phenylephrine hydrochloride) were used. High-resolution retinal imaging was performed with Spectralis OCT. The raster scan was composed of 25 B-scans covering an area of 20° × 15°. Each B-scan consisted of 512 A-lines, 6.0 mm in length, and spaced 240 μm apart. The automatic, real-time mode with active eye-tracking was used to automate eye alignment (TruTrack Active Eye Tracking; Heidelberg Engineering), improving the quality and accuracy of segmentation, and to obtain retinal scans, including 19 single horizontal axial scans in the macular area. All OCT images fulfilled the OSCAR-IB quality control criteria for retinal OCT scans. The 9-point advised protocol for OCT study terminology and elements (Advised Protocol for OCT Study Terminology and Elements [APOSTEL] recommendations) is presented in Supplemental Table 1.

**Figure 2:**
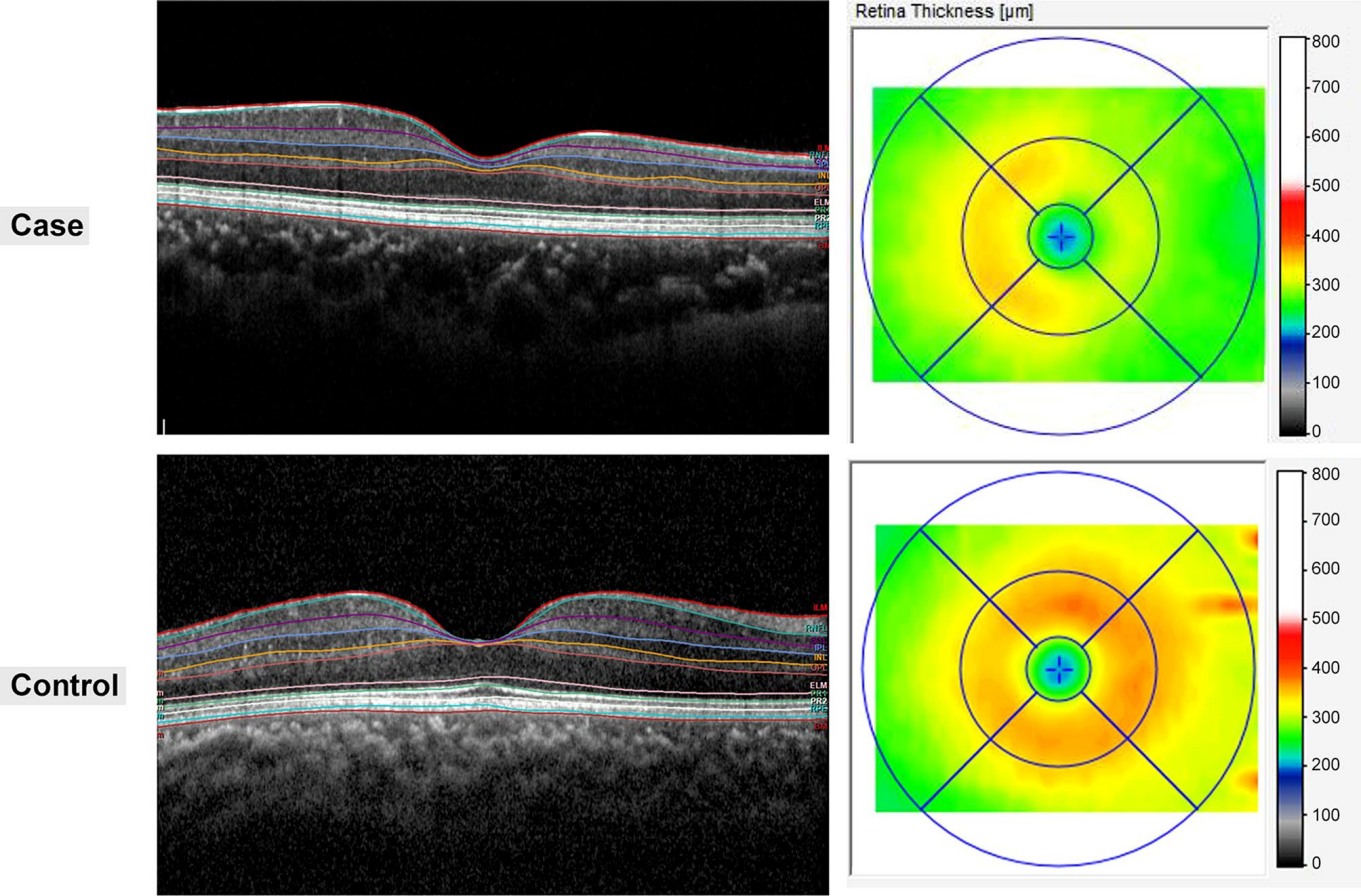
Two representative cases of patients diagnosed postoperatively with juvenile craniopharyngioma and healthy controls. B-scan of spectral-domain optical coherence tomography with auto-segmentation (in-built Spectralis mapping software, Heidelberg Eye Explorer HEYEX version 1.10.2.0) into the following retinal layers: *RNFL* retinal nerve fiber layer, *GCL* ganglion cell layer, *IPL* inner plexiform layer, *INL* inner nuclear layer, *OPL* outer plexiform layer, *ONL* outer nuclear layer, *PRL* photoreceptor layer.

Seven retinal layers were identified via automated segmentation using Spectralis software with manual correction as needed. The thickness of each layer between the vitreoretinal interface and the outer border of the retinal pigment epithelium was measured automatically. The whole retinal layer was segmented into seven respective retinal layers: the RNFL, the GCL, the IPL, the INL, the OPL, the ONL, and the PRL. Following the automated segmentation of each retinal layer, the thickness of each retinal layer in the 3- and 6-mm subfields as defined by the ETDRS grid was automatically measured using the Spectralis mapping software (Fig. 2). The average thickness of the four macular quadrants measured within the 3 mm and 6 mm zones was calculated for each layer. The segmentation was based on a validated algorithm used to measure the average thickness values within a 3 × 3 mm circle and a 6 × 6 mm extended area centered at the fovea. The foveal region, consisting of a 1 × 1 mm circle, was excluded from the analysis. The quality of all images was reviewed by two independent graders (G.I.L. and K.A.P.). We manually corrected any errors in the automated segmentation, as reported previously by Oberwahrenbrock et al.^34^.

### Statistical analysis

The demographic and clinical characteristics of the CP patients and healthy controls are presented as standard descriptive summaries (e.g., means and standard deviations for continuous variables such as age, and percentages for categorical variables such as gender). The BCVA was converted to a logarithmic scale (logMAR). The chi-squared test was used to compare categorical variables such as gender between the groups. The Mann–Whitney U test was used to compare clinical characteristics such as age, SE, BCVA, and peripapillary RNFL thickness between the group of patients and the healthy controls. The intraretinal layer thickness of the patients was compared to that of the healthy controls using linear regression analysis adjusting for age and SE. We divided all CP patients into two groups of the worse eye and better eye and performed subgroup analysis using the paired t-test and independent t-test. Bonferroni’s correction for multiple comparisons was applied to the *p-*values by multiplying the uncorrected *p-*values by 28 in the main anlaysis and the subgroup analysis. Pearson’s and Spearman’s correlation coefficients were calculated to analyze the association between intra-retinal layer thickness and functional parameters and peripapillary RNFL thickness. A *p*-value of less than 0.05 was considered statistically significant. All statistical analyses were performed with SAS version 9.4 (SAS Institute, Cary, NC, USA).

## Supplementary Information

Supplementary Information

## Data Availability

The datasets generated during and/or analyzed during the current study are available from the corresponding author upon reasonable request.

## References

[1] Muller HL (2013). Childhood craniopharyngioma. Pituitary.

[2] Bunin GR (1998). The descriptive epidemiology of craniopharyngioma. J. Neurosurg..

[3] Ohmori K, Collins J, Fukushima T (2007). Craniopharyngiomas in children. Pediatr. Neurosurg..

[4] Haupt R (2006). Epidemiological aspects of craniopharyngioma. J. Pediatr. Endocrinol. Metab..

[5] Rosemberg S, Fujiwara D (2005). Epidemiology of pediatric tumors of the nervous system according to the WHO 2000 classification: A report of 1195 cases from a single institution. Childs Nerv. Syst..

[6] Muller HL (2010). Childhood craniopharyngioma: Current concepts in diagnosis, therapy and follow-up. Nat. Rev. Endocrinol..

[7] Drapeau A (2019). Pediatric craniopharyngioma. Childs Nerv. Syst..

[8] Yano S (2016). Quality of life and clinical features of long-term survivors surgically treated for pediatric craniopharyngioma. World Neurosurg..

[9] Banc A, Stan C, Florian IS (2018). Optical coherence tomography as a marker of vision in children with optic pathway gliomas. Child's Nervous Syst..

[10] Bialer, O. Y., Goldenberg-Cohen, N., Toledano, H., Snir, M. & Michowiz, S. Retinal NFL thinning on OCT correlates with visual field loss in pediatric craniopharyngioma. *Can. J. Ophthal.* **48**, 494–499 (2013). 10.1016/j.jcjo.2013.05.001 24314410

[11] Drexler W, Fujimoto JG (2008). State-of-the-art retinal optical coherence tomography. Prog. Retinal Eye Res..

[12] Tieger MG (2017). Ganglion cell complex loss in chiasmal compression by brain tumors. J. Neuro-ophthalmol..

[13] Zhu BD (2013). Retinal nerve fiber layer thickness in a population of 12-year-old children in central China measured by iVue-100 spectral-domain optical coherence tomography: The Anyang Childhood Eye Study. Invest. Ophthalmol. Vis. Sci..

[14] Chen L (2013). Retinal nerve fiber layer thickness in normal Chinese students aged 6 to 17 years. Invest. Ophthalmol. Vis. Sci..

[15] Agarwal P, Saini VK, Gupta S, Sharma A (2014). Evaluation of central macular thickness and retinal nerve fiber layer thickness using spectral domain optical coherence tomography in a tertiary care hospital. J. Curr. Glaucoma Pract..

[16] Rajjoub RD, Trimboli-Heidler C, Packer RJ, Avery RA (2015). Reproducibility of retinal nerve fiber layer thickness measures using eye tracking in children with nonglaucomatous optic neuropathy. Am. J. Ophthalmol..

[17] Avery RA (2015). Longitudinal change of circumpapillary retinal nerve fiber layer thickness in children with optic pathway gliomas. Am. J. Ophthalmol..

[18] Avery RA (2011). Retinal nerve fiber layer thickness in children with optic pathway gliomas. Am. J. Ophthalmol..

[19] Gu S, Glaug N, Cnaan A, Packer RJ, Avery RA (2014). Ganglion cell layer-inner plexiform layer thickness and vision loss in young children with optic pathway gliomas. Invest. Ophthalmol. Vis. Sci..

[20] Yang L, Qu Y, Lu W, Liu F (2016). Evaluation of macular ganglion cell complex and peripapillary retinal nerve fiber layer in primary craniopharyngioma by fourier-domain optical coherence tomography. Med. Sci. Monit. Int. Med. J. Exp. Clin. Res..

[21] Sandvik U, Ohlsson M, Edstrom E (2019). Vascular complications in pediatric craniopharyngioma patients: A case-based update. Child's Nervous Syst..

[22] Mediero S, Noval S, Bravo-Ljubetic L, Contreras I, Carceller F (2015). Visual outcomes, visual fields, and optical coherence tomography in paediatric craniopharyngioma. Neuroophthalmology.

[23] Wan MJ (2018). Long-term visual outcomes of craniopharyngioma in children. J. Neurooncol..

[24] Perry A (2018). Pediatric pituitary adenoma: Case series, review of the literature, and a skull base treatment paradigm. J. Neurol. Surg. B Skull Base.

[25] Chen C, Okera S, Davies PE, Selva D, Crompton JL (2003). Craniopharyngioma: A review of long-term visual outcome. Clin. Exp. Ophthalmol..

[26] Moon CH, Hwang SC, Kim BT, Ohn YH, Park TK (2011). Visual prognostic value of optical coherence tomography and photopic negative response in chiasmal compression. Invest. Ophthalmol. Vis. Sci..

[27] Costello F (2013). The afferent visual pathway: Designing a structural-functional paradigm of multiple sclerosis. ISRN Neurol..

[28] Vanburen JM (1963). Trans-synaptic retrograde degeneration in the visual system of primates. J. Neurol. Neurosurg. Psychiatry.

[29] Akashi A (2014). The detection of macular analysis by SD-OCT for optic chiasmal compression neuropathy and nasotemporal overlap. Invest. Ophthalmol. Vis. Sci..

[30] Monteiro ML (2014). Evaluation of inner retinal layers in eyes with temporal hemianopic visual loss from chiasmal compression using optical coherence tomography. Invest. Ophthalmol. Vis. Sci..

[31] de Araujo RB (2017). Morphological and functional inner and outer retinal layer abnormalities in eyes with permanent temporal hemianopia from chiasmal compression. Front. Neurol..

[32] Lee, G. I., Son, K. Y., Park, K. A., Kong, D. S. & Oh, S. Y. Longitudinal changes in the retinal microstructures of eyes with chiasmal compression. *Neurology* (2020). 10.1212/WNL.0000000000011087 33093228

[33] Bender R, Lange S (2001). Adjusting for multiple testing–when and how?. J. Clin. Epidemiol..

[34] Oberwahrenbrock T (2018). Multicenter reliability of semiautomatic retinal layer segmentation using OCT. Neurol. Neuroimmunol. Neuroinflamm..

