# Supplementary material for: Inner and outer retinal layer thickness alterations in pediatric and juvenile craniopharyngioma
Source: Sci Rep. 2021 Feb 2;11:2840. doi: 10.1038/s41598-021-82107-5 (PMC7854727; doi:10.1038/s41598-021-82107-5)
Supplement: Supplementary file 1 — Supplementary Information [file 41598_2021_82107_MOESM1_ESM.docx]

**Inner and outer retinal layer thickness alterations in pediatric and juvenile craniopharyngioma**

Ga-In Lee^1^*, Kyung-Ah Park^1^*, Sei Yeul Oh^1^, Doo-Sik Kong^2^, and Sang Duk Hong^3^

Institutional affiliation:

^1^Department of Ophthalmology, Samsung Medical Center, Sungkyunkwan University School of Medicine, Seoul, Korea

^2^Department of Neurosurgery, Endoscopic Skull Base Surgery Clinic, Brain Tumor Center, Samsung Medical Center, Sungkyunkwan University School of Medicine, Seoul, Korea

^3^Department of Otorhinolaryngology-Head and Neck Surgery, Samsung Medical Center, Sungkyunkwan University School of Medicine, Seoul, Korea

***These two authors contributed equally to this work.**

Address correspondence and reprint requests to

**Corresponding author**

**Sei Yeul Oh, MD, PhD**

Professor,

Department of Ophthalmology,

Samsung Medical Center, Sungkyunkwan University School of Medicine,

81 Irwon-ro, Gangnam-gu, Seoul 06351, South Korea

Tel: (+82)-2-3410-3562, Fax: (+82)-2-3410-0074

E-mail: [syoh@skku.edu](mailto:syoh@skku.edu)

Supplemental Table 1. APOSTEL criteria

Nine-point Advised Protocol for OCT Study Terminology and Elements checklist

| **Item** | **Recommendation** |
| --- | --- |
| 1 Study Protocol | Describe how many OCT operating sites and graders were included: **one site, two graders** |
|  | Report the timing of OCT compared to other measurements (same day, delayed): **Same day** |
|  | Describe the inclusion and exclusion criteria: **Described on p. 12, lines 10- p.13, line 5.** |
| 2 Acquisition Device | For all OCT devices used, report data on: |
|  | Manufacturer: **Heidelberg Engineering, Heidelberg, Germany** |
|  | Model: **Spectralis OCT** |
|  | Software version: **Heidelberg Eye Explorer HEYEX 1.10.2.0** |
| 3 Acquisition Settings | Clearly describe the settings in which OCT scans were obtained: |
|  | Room light conditions: **dimly lit room** |
|  | Pupils dilated before examination: **yes** |
|  | Number of operators and devices: **two same devices, two professional operators** |
| 4 Scanning protocol | Clearly describe the scanning protocol, including: |
|  | Type of scan (circular, volume, star, line, other): **macular volume scan** |
|  | Location (area of interest, macula, optic nerve head papillomacular bundle, other?): **macula** |
|  | Scan parameters (with or without eye tracking): **with eye tracking** |
|  | Volume scan: size of scan area (degrees or millimeters), number of B-scans, alignment of B-scans, number of A- scans per B-scan  **Described on p 14, lines 2-4** |
|  | Radial scan: size of scan area (degrees of millimeters), number of B-scans, alignment of B-scans, number of A- scans per B-scan **N/A** |
|  | Ring scan: diameter, A-scan/B-scan, manual or automatic placement of ring or method of centering, depth resolution **N/A** |
|  | Line scan: angle, location, number of A-scans, depth resolution **N/A** |
| 5 Funduscopic imaging | Report other imaging modalities used in addition to OCT (fundoscopy, confocal scanning laser ophthalmoscopy, retinal angiography, autofluorescence imaging): **N/A** |
|  | Describe acquisition protocol including**: N/A** |
|  | Excitation wavelength |
|  | Filter sets |
|  | Number of frames averaged (if applicable) |
| 6 Postacquisition data | Describe image selection process, including: |

| Selection | |
| --- | --- |
| Quality control criteria: **OSCAR-IB criteria** | |
| Postacquisition discard (number and criteria): **No images were discarded** | |
| Eye selection strategy (if applicable): **We selected only a single eye showing the worse visual acuity or VF defects was selected for the analysis, but if visual acuity was less than 20/200 in the eye with worse VF defects, the other eye with visual acuity greater than 20/200 was selected for more accurate OCT examination with better cooperation. in each patient, if the quality of image is inappropriate, the scan with the best image quality was included in the analysis.** | |
| 7 Postacquisition analysis | Describe all postacquisition steps: |
| Software used for processing scans and segmentation (may be different from acquisition software): **Using built-in software of the Spectralis with the autosegmentation algorithm (Heidelberg Eye Explorer software), described on p 14, lines 17-20** | |
| Which individual retinal layers were segmented/included:  **RNFL, GCL, IPL, INL, OPL, ONL, PRL** | |
| Method of segmentation (automated, semiautomated, or manually): **automated, described on p 14, lines 12-15** | |
| How potential bias was addressed in the case of manual segmentation (masking): **The OCT grader was masked to participant group assignment.** | |
| 8 Nomenclature and abbreviations | Define: |
|  | Anatomical structures analyzed: **RNFL, GCL, IPL, INL, OPL, ONL, PRL, peripapillary RNFL thickness** |
|  | Units of provided measurements (e.g., volume or thickness):  **thickness in μm** |
| 9 Statistical approach | Describe: |
|  | Statistical models used for the analyses of OCT data: **Described on p 15, lines 14-15** |
|  | Whether data were analyzed by eye or by patient: **Data were analyzed by patient with using only on eye.** |
